# The absence of *luxS* reduces the invasion of *Avibacterium paragallinarum* but is not essential for virulence

**DOI:** 10.3389/fvets.2024.1427966

**Published:** 2024-08-27

**Authors:** Donghai Li, Caiyun Huo, Guiping Li, Menghan Zhu, Fuzhou Xu, Jian Qiao, Huiling Sun

**Affiliations:** ^1^Department of Pathophysiology, College of Veterinary Medicine, China Agricultural University, Beijing, China; ^2^Beijing Key Laboratory for Prevention and Control of Infectious Diseases in Livestock and Poultry, Institute of Animal Husbandry and Veterinary Medicine, Beijing Academy of Agriculture and Forestry Sciences, Beijing, China

**Keywords:** quorum sensing, LuxS, autoinducer-2, *Avibacterium paragallinarum*, virulence

## Abstract

The contagious respiratory pathogen, *Avibacterium paragallinarum*, contributes to infectious coryza in poultry. However, commercial vaccines have not shown perfect protection against infectious coryza. To search for an alternative approach, this research aimed to investigate whether the quorum-sensing system of pathogens plays a crucial role in their survival and pathogenicity. The LuxS/AI-2 quorum-sensing system in many Gram-negative and Gram-positive bacteria senses environmental changes to regulate physiological traits and virulent properties, and the role of the *luxS* gene in *Av. paragallinarum* remains unclear. To investigate the effect of the *luxS* gene in the quorum-sensing system of *Av. paragallinarum*, we constructed a *luxS* mutant. Bioluminescence analysis indicated that the *luxS* gene plays a vital role in the LuxS/AI-2 quorum-sensing system. The analysis of the LuxS/AI-2 system-related genes showed the level of *pfs* mRNA to be significantly increased in the mutant strain; however, *lsrR*, *lsrK*, and *lsrB* mRNA levels were not significantly different compared with the wild type. The ability of the *luxS* mutant strain to invade HD11 and DF-1 cells was significantly decreased compared with the wild-type strain. In addition, all chickens challenged with various doses of the *luxS* mutant strain developed infections and symptoms, and those challenged with the lowest dose exhibited only minor differences compared to chickens challenged with the wild-type strain. Thus, the deletion of the *luxS* gene reduces the invasion, but the *luxS* gene does not play an essential role in the pathogenesis of *A. paragallinarum*.

## 1 Introduction

*Avibacterium paragallinarum*, a member of the bacterial *Pasteurellaceae* family, is the etiological agent of infectious coryza, a highly contagious respiratory disease in laying hens (1, 2). Facial swelling, conjunctivitis, nasal discharge, diarrhea, and anorexia are the main clinical symptoms (3). Infectious coryza causes serious decreases in egg production and enormous economic losses for the poultry industry worldwide (4). Many of the microorganisms that reside in the respiratory tract are beneficial to the host’s normal development and physiology (5), and the bacteria with programmed therapeutic functions are now a tangible reality (6). The interactions between microbes therefore present possibilities for new strategies to treat and prevent infectious disease.

Quorum sensing is a cell-to-cell communication system by which bacteria sense their population density and other bacterial species through autoinducers (AIs) to regulate functions (7). Many Gram-negative bacteria produce N-acyl homoserine lactone signaling molecules, and the most Gram-positive bacteria use modified peptides as signal molecules. However, the enzyme, LuxS, which synthesizes the signaling molecule, AI-2, is the only quorum-sensing pathway in both Gram-positive and Gram-negative bacteria (8). Many pathogens use LuxS to exert AI-2 control over important virulence factors and essential metabolic pathways in pathogens, such as methionine synthesis and furanone production (7).

LuxS is an important enzyme in the activated methyl cycle, which is a means of methylating cellular components and recycling certain sulfur-containing amino acids in bacteria, archaea, and eukaryotes. A component of the activated methyl cycle, S-adenosyl-l-homocysteine, is detoxified to S-ribosyl-homocysteine by the Pfs enzyme, 5′-methylthioadenosine/s-adenosylhomocysteine nucleosidase. S-ribosyl-homocysteine, a substrate of LuxS, is catabolized to homocysteine, and 4,5-dihydroxy-2,3-pentanedione (the precursor of AI-2) (9, 10). In *S. typhimurium* and *E. coli*, AI-2 is recognized and bound by the LsrB protein, and then the AI-2 is phosphorylated by LsrK (11, 12). The phosphorylated form of AI-2 induces *lsr* transcription, and the process is acted upon by binding to LsrR, the repressor of the *lsr* operon (13). The LuxS/AI-2 quorum-sensing system is present in many *Pasteurellaceae* strains, and the system is associated with bacterial biofilms, cell adhesion, motility, and virulence (14–17). However, the role of the LuxS in *Av. paragallinarum* is unknown.

The involvement of LuxS in both the quorum-sensing system and the activated methyl cycle implies that it is important to study the function of LuxS in the *in vivo* pathogenicity of *Av. paragallinarum*. Here, we constructed a *luxS* deletion mutant of *Av. paragallinarum* to investigate the LuxS/AI-2 activity. We also determined differences in the expression of quorum sensing-related genes between the wild-type and the mutant strain. Furthermore, bacterial adherence and invasion were evaluated in the *luxS* knockout mutant. Finally, a pathogenicity analysis of the *luxS* mutant strain was performed.

## 2 Materials and methods

### 2.1 Bacterial strains, primers, and culture conditions

A clinical isolate of *Av. paragallinarum* strain 3005 (serogroup C) was isolated in China in 2018 (18). Wild-type, mutant, and complemented strains were grown in tryptic soy broth or tryptic soy agar medium supplemented with 10% chicken serum and 0.0025% nicotinamide adenine dinucleotide (NAD) at 37°C, and the agar plates were cultured under 5% carbon dioxide. *Vibrio harveyi* strains BB170 and BB152 were supplied by Professor Han (The Chinese Academy of Agricultural Sciences, Shanghai, China) and cultivated in a modified autoinducer bioassay (AB) medium at 30°C (19). *Escherichia coli* DH5α and BL21 (DE3) (Vazyme, Nanjing, China) were grown routinely in Lennox broth or on a solid medium containing 1.5% agar at 37°C. All primers used are listed in Table 1.

**Table 1:** Primers used in this study.

Primers	Oligonucleotide sequence (5′–3′)	Description	Product size (bp)	References
pET32a-luxS-F	GGCTGATATCGGATCCatgccgttacttgatagttttaaagtcg	*luxS* fragment for construction pET32a-LuxS plasmid	507	This study
pET32a-luxS-R	GCTCGAGTGCGGCCGCttattgtgcaagcaaacgctc			
luxS-pET32a-F	caagtaacggcatGGATCCGATATCAGCCATGG	expression vector fragment for construction pET32a-LuxS plasmid	5,875	This study
luxS-pET32a-R	gcttgcacaataaGCGGCCGCACTCGAGCACCA			
luxS-UF	CGGGATTTGCTCATAAGCCAA	the total fragment: *luxS*, *luxS* upstream, and *luxS* downstream	2,982	This study
luxS-UR	TGCTCGTATTGCCTCCGTTAA			
CM-F	TGCTCGGCGGTGTTCCTTTCC	chloramphenicol cassette to replace *luxS* gene	801	(20)
CM-R	GCGCCCTTTAGTTCCTAAAGG			
luxS-F	ATGCCGTTACTTGATAGTTTTAAAG	Cloned into T Vector pM19 to establish the complemented plasmid	507	This study
luxS-R	TTATTGTGCAAGCAAACGC			
rpoB-F	GCTTAATGCCGCTTCACCTA	Housekeeping gene	131	(21)
rpoB-R	AGCGTGTGGTGCAAGAAGAT			
RT-pfs-F	GGGTTGATTTGCTCAGGGGA	the sole intracellular source of the substrate of *luxS*	130	This study
RT-pfs-R	TGAAAGCGTGGCAAACTTGA			
RT-lsrR-F	CAGCAACGAGGTCAAGCAAC	encoding a repressor for the *luxS*-reguated *lsr* operon	163	This study
RT-lsrR-R	CGCTGATCAATTCCTCGTGC			
RT-lsrB-F	CTACGATGGACCGACCGAAC	AI-2-binding protein gene	145	This study
RT-lsrB-R	GCCTCGTTTCATTGCTCGTT			
RT-lsrK-F	TGGTCGCCTGCATTACTTGA	encoding a kinase responsible for AI-2 phosphorylation	153	This study
RT-lsrK-R	ATCACCACCACCGACAACAA			

Lower case sequence denotes homologous linking sequence used in plasmid construction.

### 2.2 The expression of LuxS and production of LuxS antiserum

Primers pET32a-luxS-F/R and luxS-pET32a-F/R were used to amplify the *luxS* fragment from *Av. paragallinarum* strain 3005 and the expression vector from pET-32a, respectively. The PCR products were used to construct the pET32a-luxS expression plasmid using the Hieff Clone^®^ Plus One Step Cloning Kit (Yeasen, Shanghai, China). *Escherichia coli* BL21 (DE3) transformed with the pET32a-luxS plasmid was used to express recombinant LuxS protein (His-LuxS). His-LuxS was purified using a His-tagged protein purification kit (CWbio, Jiangsu, China) and analyzed by sodium dodecyl sulfate-polyacrylamide gel electrophoresis (SDS-PAGE) and Western blotting (22).

The His-LuxS protein was mixed with ‘QuickAntibody,’ a water-soluble immune adjuvant (Biodragon, Beijing, China), to prepare mouse antiserum. The His-LuxS protein was diluted to 1 mg/mL, and then 50 μL of protein was quickly mixed with the immune adjuvant by 1:1. Six male BALB/c mice were subcutaneously immunized with the mixture at a dose of 50 μg/mouse two times, with an interval of 14 days. Blood was collected from the tail vein, and immune serum titers were assessed by enzyme-linked immunosorbent assays (ELISAs). Mice with high antibody titers were euthanized in 30% CO_2_ cages, and their sera were collected.

### 2.3 Construction of luxS mutant and complemented strains

A fragment consisting of the *luxS* gene and *luxS* upstream and *luxS* downstream was amplified from the *Av. paragallinarum* strain 3005 using primers luxS-UF/luxS-UR and cloned into T Vector pMD19 (Takara, Dalian, China). The fragment was then inserted into the pGEM^®^-T Easy vector using *Hin*dIII and *Eco*RI restriction enzyme sites. The *luxS* gene of the obtained pGEM^®^-T Easy vector was replaced with a chloramphenicol cassette to generate the recombinant plasmid. The recombinant plasmid was introduced into *Av. paragallinarum* by electroporation (23). Briefly, wild-type *Av. paragallinarum* was washed three times with 9.3% (wt/vol) of cold sucrose, and 65 μL aliquots were mixed with recombinant plasmid (0.5 μg) and transferred to precooled electroporation cuvettes (gap size, 1 mm). After electrotransformation, the positive strain was selected on tryptic soy agar containing 10 μg/mL of chloramphenicol.

The complemented strain was constructed using the pBBR1MCS-2 vector as described, with a simple modification for the construction of the *luxS* mutant. The *luxS* gene was amplified using luxS-F/luxS-R primers and then cloned into T Vector pMD19. *Sal*I and *Xba*I restriction enzymes were then used on the T-Vector pMD19 and pBBR1MCS-2 to establish the complemented plasmid. The plasmid was introduced into the *luxS* mutant strain by electrotransformation, and 50 μg/mL of kanamycin was used to select the complemented strain.

Complementation was verified by agarose gel electrophoresis analysis of the *luxS* gene using luxS-F/R primers, the chloramphenicol cassette using CM-F/R primers (20), and the total fragment using luxS-UF/luxS-UR primers. The deletion of the LuxS protein was confirmed by Western blot analysis.

### 2.4 AI-2 bioluminescence assay

The assay was performed essentially as previously described, with modification (24). Briefly, the test bacteria of *Av. paragallinarum* (wild-type, mutant strain, and complemented strain) was grown on tryptic soy agar plates and then washed with the AB medium. The washed medium and *Vibrio harveyi* strains BB152 in the logarithmic phase of growth were centrifuged at 12,000 *g* for 5 min, and the supernatants were collected and filtered through a 0.22 μm filter (Millipore, Billerica, USA). The *V. harveyi* BB170 reporter strain was grown in an orbital shaker for 16 h at 30°C. The cultured suspension was then diluted 1:5,000 in fresh AB medium. In total, 20 μL of filtered fluid were mixed with 180 μL of diluted BB170 in a flat-bottomed 96-well microtiter plate (Corning, New York, USA), followed by shaking at 30°C, 180 rpm for 6 h. Notably, 10% (v/v) filtered *V. harveyi* BB152 fluid was used as a positive control, and 10% (v/v) sterile PBS (0.01 M, pH = 7.4) was used as a negative control. Luminescence was measured every hour using a Synergy H1 microplate reader (BioTeK SYNERGY H1, Vermont, USA).

### 2.5 Detection of mRNAs of related quorum sensing genes

Total RNA was extracted from bacteria using a GenJET RNA Purification Kit (Thermo, Waltham, USA) following the manufacturer’s protocol and confirmed using a NanoDrop1000 spectrophotometer (NanoDrop Technologies Inc., Waltham, USA). RNA samples with a 260/280 nm ratio between 1.9 and 2.1 were used to synthesize cDNA using an EasyScript First-Strand cDNA Synthesis SuperMix kit (Trans, Beijing, China) according to the manufacturer’s instructions. The cDNA samples were stored at −20°C for later use.

Real-time quantitative PCR (RT-qPCR) was performed using SYBR Green SuperReal premix plus (Tiangen, Beijing, China). Total sample volumes of 25 μL contained 12.5 μL of PCR Master mix, 1 μL of DNA (or water as a negative control), 0.5 μL of forward/reverse primer, and 10.5 μL of water. Each reaction was performed in triplicate. To measure related quorum sensing genes, including *pfs*, *lsrR*, *lsrB*, *lsrK*, and *rpoB* (as a housekeeping control) (21), RT-qPCRs were performed with an initial denaturation step of 95°C for 15 min, followed by 40 cycles at 95°C for 10 s and 55°C for 30 s on a Bio-Rad IQ5 (Bio-Rad, Hercules, USA) (21, 25).

### 2.6 Bacterial adherence and invasion assays

The effects of the *Av. paragallinarum luxS* gene on cell adhesion and invasion were evaluated in assays using chicken macrophage (HD11, this laboratory) and chicken embryo fibroblast (DF-1, this laboratory) cells following a previously described method (26). Briefly, cells were seeded onto 24-well cell culture plates at 90% confluence. The wells were washed three times with PBS and infected with *Av. paragallinarum* (1 × 10^7^ CFU/well) or its *luxS* mutant (1 × 10^7^ CFU/well) in DMEM/F12 medium containing 10% (v/v) fetal bovine serum for HD11 cultures and DMEM medium containing 10% (v/v) fetal bovine serum for DF-1 cultures. After incubation at 37°C in 5% CO_2_ for 1 h, cells were rigorously washed with PBS three times to remove non-adherent bacteria and then incubated with 100 μL of 0.25% trypsin/EDTA containing 0.5% Triton X-100 for 10 min. The resulting cell suspension was serially diluted 10-fold with PBS and dispersed onto tryptic soy agar plates containing NAD and chicken serum. Finally, the number of adherent bacteria was counted.

To assess the effect of the *Av. paragallinarum luxS* gene on cell invasion, cell culture and infection were performed as described for the bacterial adherence assay. The infected cells were incubated for 3 h and 6 h. Following washing with PBS three times, 100 μg/mL of cefalexin and 50 μg/mL of kanamycin were added to each well and incubated at 37°C in 5% CO_2_ for 3 h to kill the extracellular bacteria. Wells were then treated with 100 μL of 0.25% trypsin/EDTA containing 0.5% Triton X-100 for 10 min, and the invaded bacteria were counted on tryptic soy agar plates containing NAD and chicken serum. Assays were performed for all treatment groups in duplicate three times.

### 2.7 Transmission electron microscopy (TEM)

TEM was used to visualize the *in vitro* invasion of cells by wild-type and *luxS* mutant *Av. paragallinarum*. DF-1 and HD11 cells were cultivated in culture dishes (10 cm diameter). The cell culture and infection were performed as described for the bacterial invasion assay. After pathogen infection for 6 h and antibiotic treatment for 3 h, the culture dishes were washed with PBS three times. The cells were then scraped from the bottom of the dishes with a cell scraper and collected in 1.5 mL sterile centrifuge tubes. Collected cells were fixed with 1 mL of fixative solution (2.5% glutaraldehyde) for 48 h. Ultrathin sections were then made and stained with 0.1% uranyl acetate and 0.1% lead citrate for transmission electron microscopy (Hitachi H-7500, Chiyoda, Japan).

### 2.8 Animal challenges

To assess potential changes in virulence associated with disrupting the *luxS* gene, wild-type and mutant *Av. paragallinarum* were used in chicken models (27). We carried out the animal experiments in strict accordance with the requirements of the Laboratory Animal Requirements of Environment and Housing Facilities (GB14925-2010, the National Laboratory Animal Standardization Technical Committee) and the Chinese Regulations of Laboratory Animals Guidelines (Ministry of Science and Technology of People’s Republic of China). All experimental procedures were approved and audited by the Beijing Academy of Agriculture and Forestry Sciences Animal Care and Use Committee guidelines [ID: SYXK (Jing) 2023–0005], which were approved by the Animal Welfare Committee of the Beijing Academy of Agriculture and Forestry Sciences (10 January 2023). Five-week-old male specific pathogen free White Leghorn chickens were randomly divided into seven experimental groups (*n* = 6 per group). The *luxS* mutant strain and wild strain infected three groups of chickens with doses of 1.5 × 10^5^ CFU, 1.5 × 10^4^ CFU, and 1.5 × 10^3^ CFU, respectively. Each bird was challenged by infraorbital sinus inoculation with 0.2 mL of diluted bacteria. The control group was treated with PBS only. The clinical signs of nasal discharge and facial swelling in the challenged chickens were used to assess the morbidity of the chickens. According to a previously reported scoring system (28), 0 represents no clinical signs; 1 represents mild clinical signs (slight facial swelling); 2 represents moderate clinical signs (moderate facial swelling and nasal discharge); and 3 represents severe clinical signs (severe facial swelling, abundant nasal swelling, lacrimation, and partially or completely closed eye).

## 3 Results

### 3.1 Preparation of the mouse anti-LuxS antiserum

**Figure 1:**
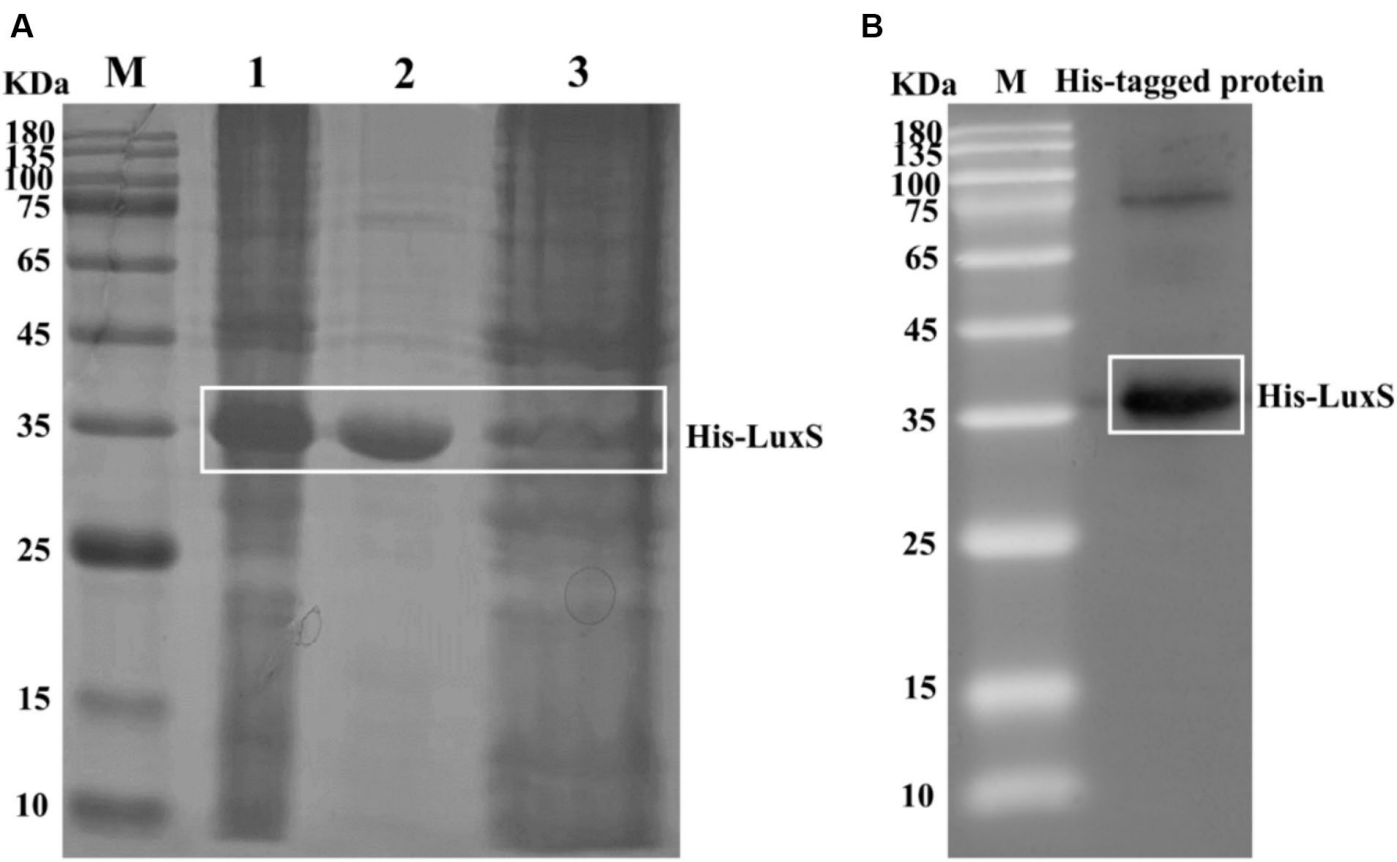
Induction of recombinant His-LuxS expression. The protein in extracts was obtained after the lysis of cells from induced *E. coli* BL21. SDS-PAGE analysis of the products expressed by *E. coli* BL21 harboring pET32a-LuxS **(A)**. Lane 1, pET32a-LuxS/BL21 isopropyl β-d-1-thiogalactopyranoside induced; lane 2, the purified recombinant His-LuxS protein; lane 3, Ni-affinity filtered solution. Western blot analysis of the recombinant protein using an anti-His tag antibody **(B)**.

We cloned the *luxS* gene into pET32a and successfully expressed a recombinant His-LuxS fusion protein in *E. coli* BL21. The purified protein was a distinct band on SDS-PAGE analysis (Figure 1A). A His antibody was used to confirm the His-tag on the purified protein by Western blot analysis (Figure 1B). A mouse anti-LuxS antiserum diluted 10,000 times was assessed by ELISA and the antibody titer OD450 > 1.0.

### 3.2 Construction and verification of the LuxS mutant strain

**Figure 2:**
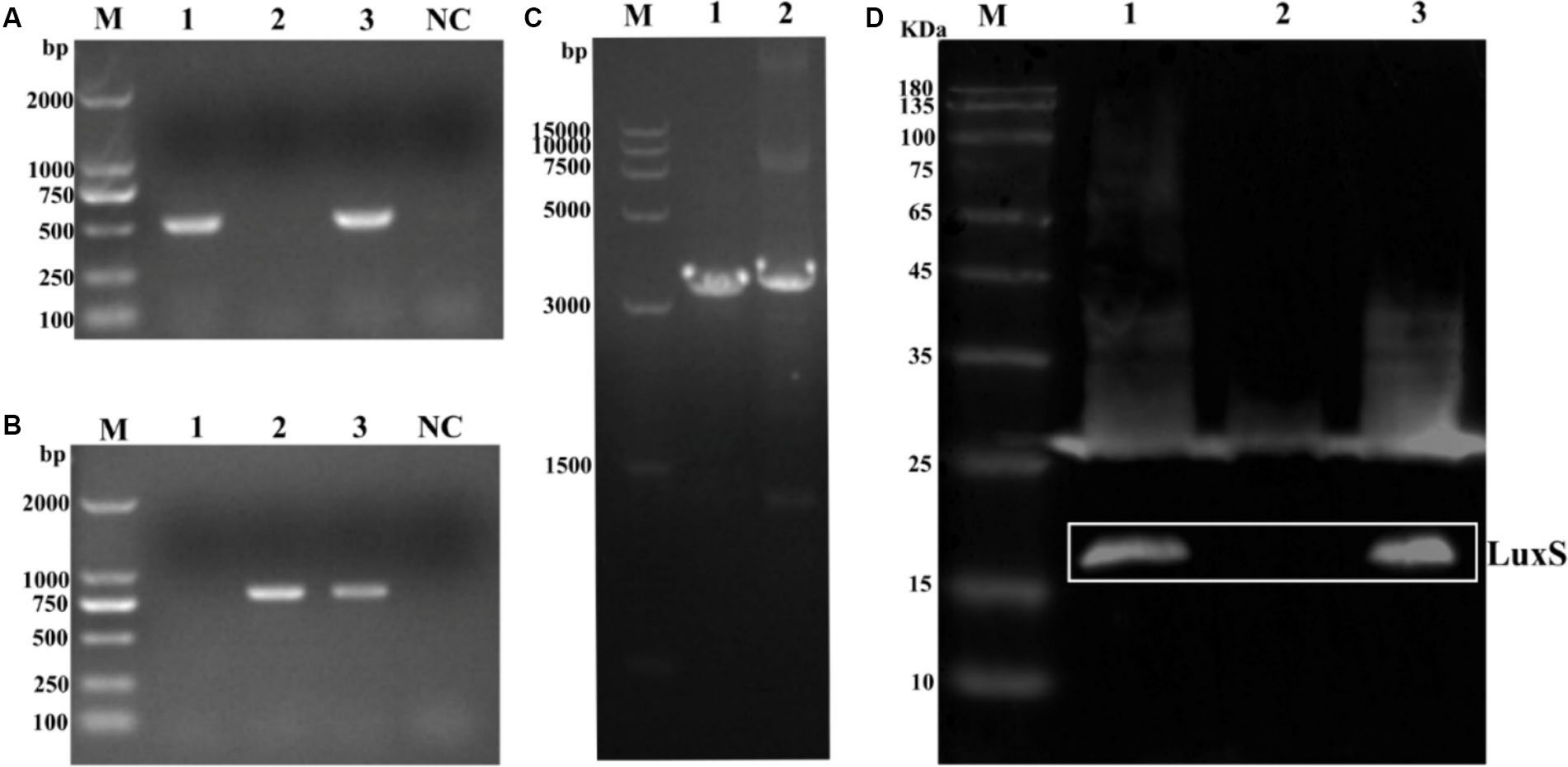
Identification of mutant strain and complemented strains of *Av. paragallinarum*. Agarose gel electrophoresis analysis of the *luxS* gene **(A)**, chloramphenicol cassette **(B)**, and the total fragments [**(C)**, the chloramphenicol cassette is longer (294 bp) than the *luxS* sequence]. Western blot analysis of the LuxS protein deletion **(D)**. The LuxS is 17 kDa. Lane 1, wild-type; lane 2, mutant strain; lane 3, complemented strain; NC, negative control.

Deletion of the *luxS* gene and complementation of the *luxS* gene were confirmed by PCR (Figure 2A) using primers luxS-F/R. The *luxS* gene was replaced by a chloramphenicol cassette; therefore, the chloramphenicol cassette in *luxS* mutants was identified by PCR using CM-F/R primers (Figure 2B). To ensure the mutation site was correct, the fragments were amplified from wild-type and mutant strains (Figure 2C). The predicted size of the LuxS protein is 17 kDa. Deletion of the LuxS protein was confirmed by Western blotting, showing no band in 17 kDa of the mutant strain (Figure 2D).

### 3.3 Detection of AI-2 activity and expression of related quorum sensing genes

**Figure 3:**
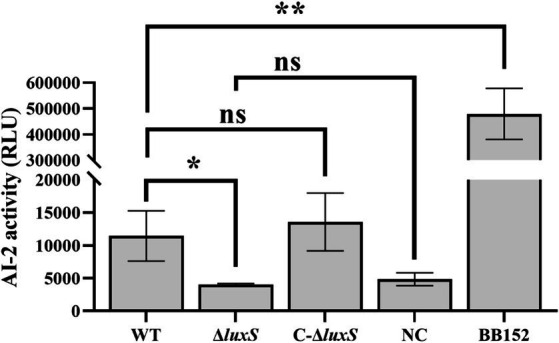
Bioluminosity values after 6 h of growth. AI-2 activity is represented by relative light units (RLU) to compare AI-2 molecule expression among strains. *Vibrio harveyi* BB152 served as a positive control, and AB medium was used as a negative control (NC). The error bars represent the standard deviation from three independent experiments. * represents *p* < 0.05, ** represents *p* < 0.01, and ns represents no significant difference.

Test supernatants of AB medium washes of lawn plates of *Av. paragallinarum* (wild-type, mutant strain, and complemented strain) were measured, and there was a significant drop of AI-2 activity in the *luxS* mutant, and the activity was successfully recovered in the complemented strain (Figure 3). The results indicated that LuxS is necessary for AI-2 production in *Av. paragallinarum*.

**Figure 4:**
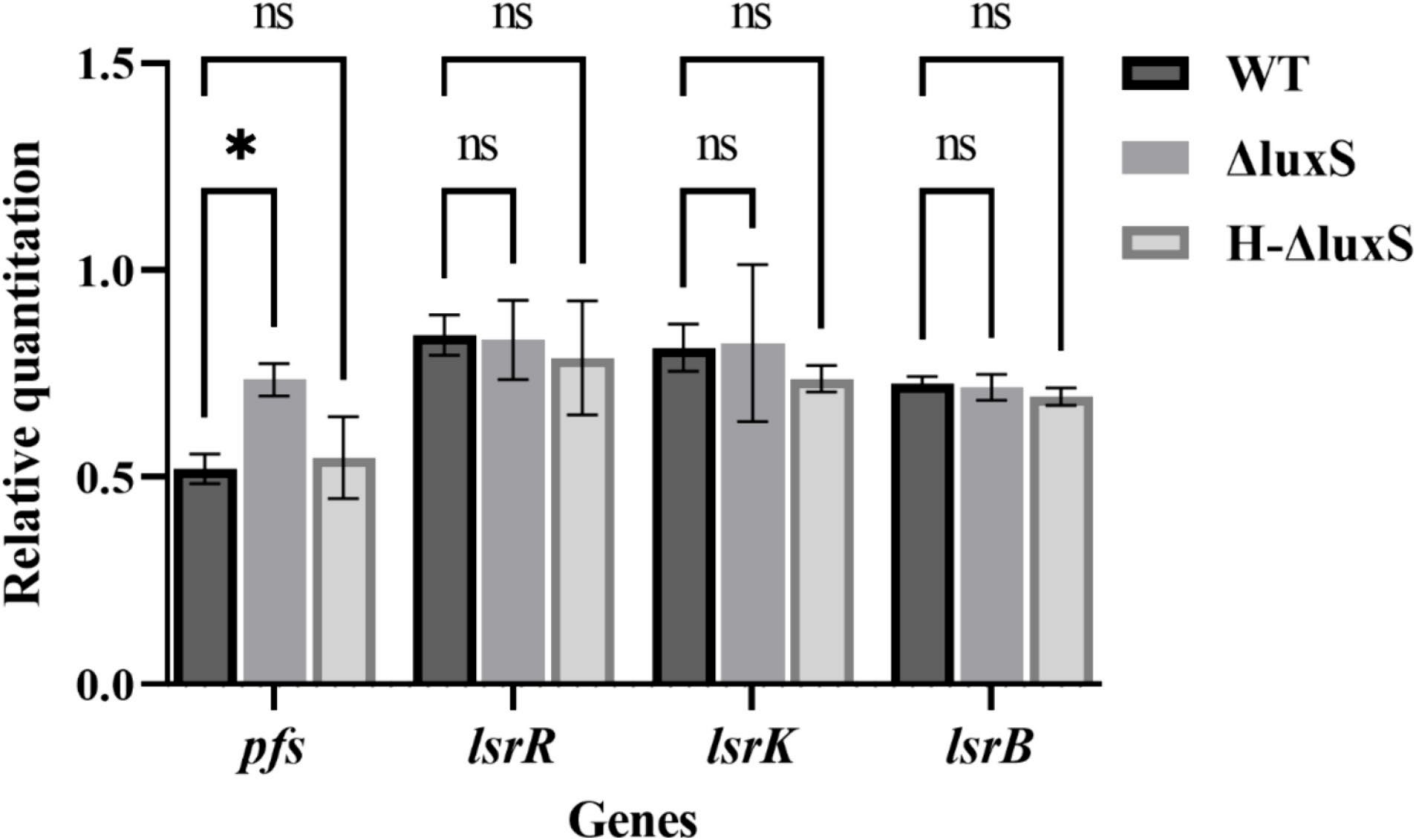
Effects of the *luxS* mutant on the expression of quorum sensing genes in *Av. paragallinarum*. qRT-PCR showed increased *pfs* expression (*p* = 0.0229) in the mutant compared with the wild-type strain, and recovered *pfs* expression to a level similar to that of the wild-type strain in the complemented strain. Other related genes did not show statistical differences in expression between the wild-type and the *luxS* mutant. * represents *p* < 0.05. Data represent means ± SD of three experiments conducted in triplicate.

In addition, real-time qPCR showed no significant differences in mRNA levels of related quorum sensing genes, *lsrR*, *lsrB,* and *lsrK*, between the wild-type strain, the mutant strain, and the complemented strain. However, the mRNA level of the *pfs* gene increased remarkably in the *luxS* mutant strain compared with the wild type, and the mRNA level of the *pfs* of the complemented strain was recovered to a level similar to that of the wild type (Figure 4). These results indicate that *Av. paragallinarum* LuxS is an important enzyme in the activated methyl cycle, but may not be involved in the conventional LuxS/AI-2 quorum-sensing system.

### 3.4 Pathogenicity of the LuxS mutant strain

**Figure 5:**
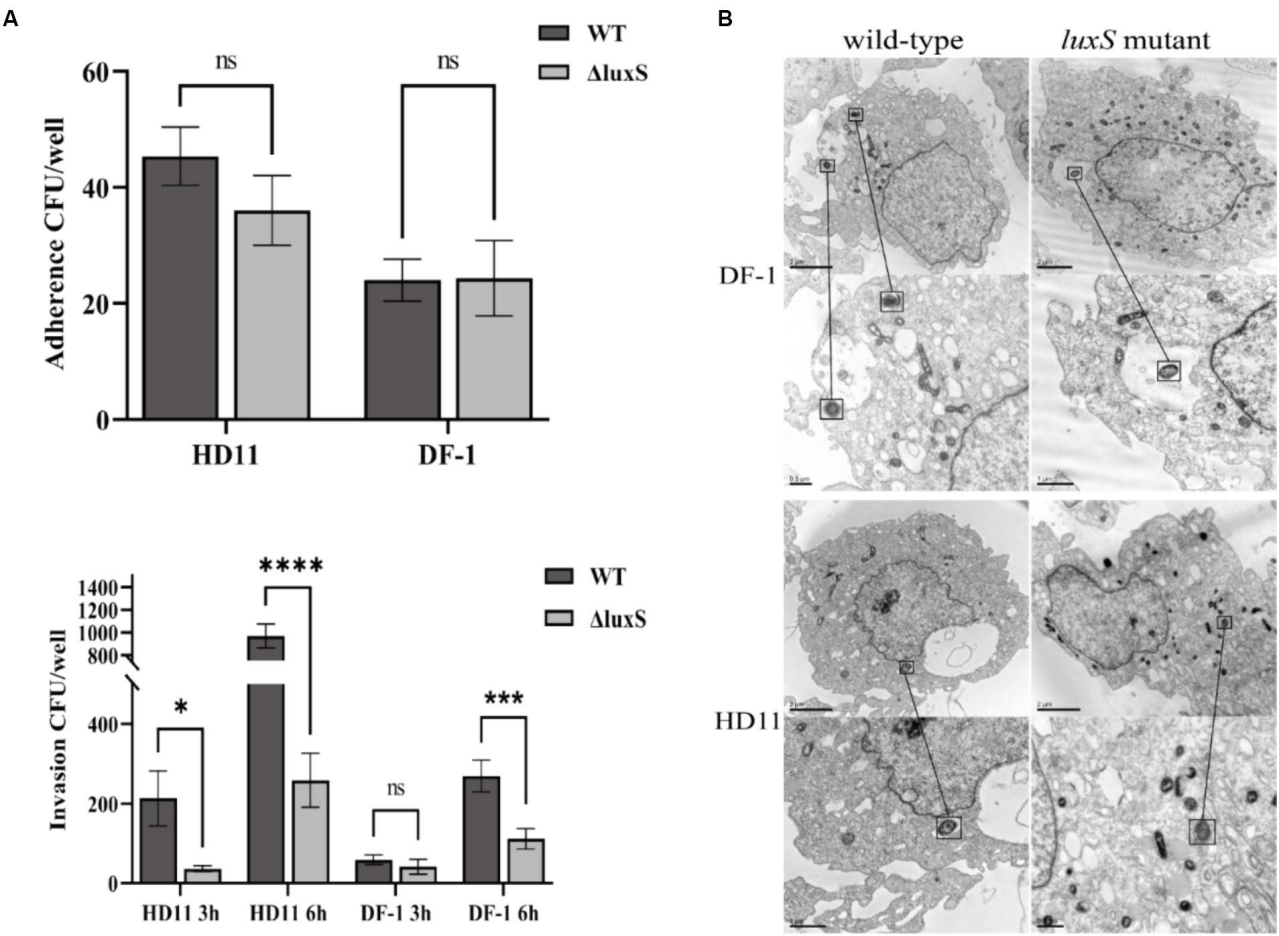
Cell adherence and invasion assay. Infection HD11 and DF-1 cells by wild-type or the *luxS* mutant. There was no statistical difference in adherence to HD11 or DF-1 cells between the wild-type and the *luxS* mutant. There was significantly reduced invasion of both HD11 and DF-1 cells by the *luxS* mutant. * represents *p* < 0.05, *** represents *p* < 0.001, **** represents *p* < 0.0001, and ns represents no significant difference. The columns represent the means and standard deviations of three experiments **(A)**. Transmission electron microscopy of DF-1 and HD11 cells shows infection with wild-type and mutant *Av. paragallinarum*, and the bacteria was noted in the black box **(B)**.

There was no distinct difference in adherence to HD11 and DF-1 cells between the wild type and the mutant. Deletion of the *luxS* gene, however, significantly reduced the capacity to invade HD11 and DF-1 cells (Figure 5A). The *Av. paragallinarum* invasion of DF-1 and HD11 cells by the wild type and the mutant was visualized by TEM (Figure 5B).

**Figure 6:**
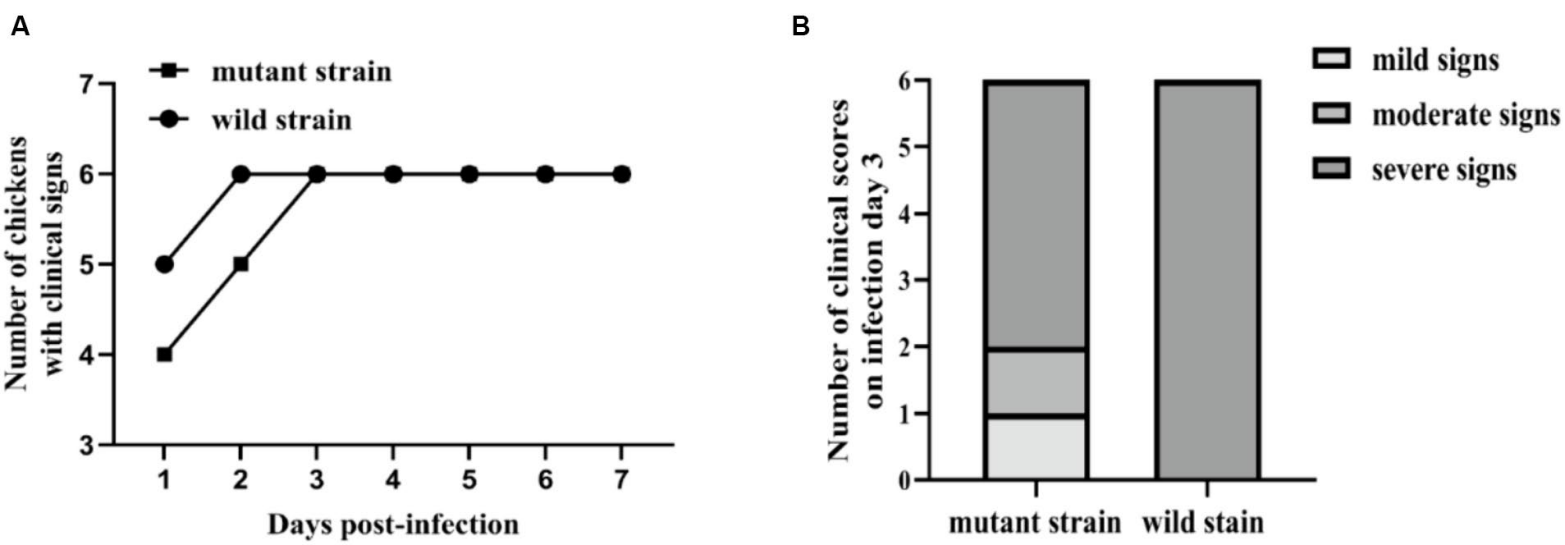
Pathogenicity tests of *luxS* mutant strain *in vivo*. Clinical signs of infectious coryza were recorded from days 1–7 post-infection. The number of animals infected with the bacteria of the lowest dose (1.5 × 10^3^ CFU) is shown in **(A)**, and the number of clinical scores was measured on infection day 3 **(B)**.

To evaluate the impact of the *luxS* gene on the virulence of *Av. paragallinarum in vivo*, we used a relatively low challenge dose to ensure the virulence of a low level of the mutant strain could be observed. Clinical signs of infectious coryza were recorded from days 1–7 post-infection. The chickens infected with wild-type and mutant strains at the dose of 1.5 × 10^5^ CFU or 1.5 × 10^4^ CFU all displayed serious facial and nasal discharge on day 1. However, there were some minor differences when the challenge dose was 1.5 × 10^3^ CFU. The chickens infected with the mutant strain showed clinical symptoms on day 3 after the challenge, while the wild strain showed symptoms on day 1 (Figure 6A). Compared with wild strain infection, the chickens infected with the mutant strain of the lowest dose (1.5 × 10^3^ CFU) showed milder clinical manifestations. One chicken displayed milder nasal discharge, one chicken showed moderate facial swelling, and four out of six chickens displayed serious facial swelling and nasal discharge, the same clinical signs as the wild strain (Figure 6B). These minor differences indicate that the *luxS* gene does not play an essential role in the pathogenesis of *Avibacterium paragallinarum*.

## 4 Discussion

LuxS is widely distributed among bacteria. It is involved in the production of autoinducer (AI-2) and is an integral component of the activated methyl cycle in bacteria (29). A large number of Gram-negative and Gram-positive bacteria are universally present in the LuxS, and AI-2 is considered an inter-species quorum sensing signal (30). Quorum sensing is a communication method among bacteria (30). In the same environment, they exchange complex and precise information by releasing and receiving tiny chemical molecules as signals. In the previous study, LuxS was associated with many virulence features, including biofilm formation, toxin production, adherence, motility, and stress response, as well as being involved in metabolisms such as iron, sulfur, and carbon metabolism (7). The family of *Pasteurellaceae* strains *Glaesserella parasuis*, *Haemophilus influenzae*, *Mannheimia haemolytica*, and *Actinobacillus pleuropneumoniae* exist in the LuxS/AI-2 system, and the *luxS* mutant affected the physiological and virulence of the strains (14, 17, 31, 32). As a member of the *Pasteurellaceae* family, the role of the LuxS in *Av. paragallinarum* remains unclear. Therefore, the main focus of this study is to investigate whether the LuxS affects the pathogenicity of *Av. paragallinarum*, as well as its impact on cell adhesion and invasion, and the regulation of other related molecules. We generated the *luxS* mutant strain and evaluated its adhesion and invasion of HD11 and DF-1 cells, while also evaluating its virulence in chickens. Similar to the previous studies showing that LuxS is associated with pathogen virulence, this study showed delayed and milder clinical symptoms between animals challenged with *luxS* mutant *Av. paragallinarum*. These results indicate that the LuxS may not play an essential role in *Av. paragallinarum* infection.

In this study, different strains of *Av. paragallinarum* were used to construct the *luxS* mutant, including standard strains 221, 0222, and Modesto and isolate 3005. However, only 3005-Δ*luxS* was obtained. Previously, the same situation was found when constructing the HutZ mutant of *Av. paragallinarum*, which is involved in iron homeostasis in *Av. paragallinarum* (20). It seems that different strains had different mutation probabilities. Two isolates were used, and the capsular mutants of *Av. paragallinarum* were constructed by the inactivation of the *hctA* gene using the TargeTronH gene knockout system (33). In addition, to verify the conservation of the *luxS* gene in *Av. paragallinarum*, several *luxS* gene sequences of *Av. paragallinarum* and other *Pasteurellaceae* strains were selected from the NCBI database, and homology analysis was performed. A phylogenetic analysis shows the genetic relationships among *Av. paragallinarum* and other *Pasteurellaceae* strains. The homology of the *luxS* gene among reference strains and local isolates of *Av. paragallinarum* is over 99% and ranges from 69 to 78% with other *Pasteurellaceae* strains (Supplementary Figure 1). This analysis shows that the *luxS* gene sequence is highly conserved among *Av. paragallinarum*. Therefore, we used strain 3,005 as a representative to study the function of LuxS. In the future, more strains will be used to verify the difference in mutant rate between standard strains and isolates.

AI-2-like molecules can be detected in many bacterial species by the reporter strain, *V. harveyi* BB170 (32, 34, 35). Certain components in tryptic soy broth may interfere with AI-2 activity (19); therefore, AB medium wash supernatants from lawn plates of *Av. paragallinarum* were used in AI-2 detection. The AI-2 detection assay showed that the LuxS/AI-2 system exists in *Av. paragallinarum* and that LuxS is a key gene in AI-2 synthesis because the luminescence value of the *luxS* deletion group was almost the same as that of the negative control. Furthermore, some regulated genes are associated with AI-2 recognition and modification; therefore, we assessed the effect of the *luxS* mutant on the transcription of *lsrB* (the AI-2-binding protein gene) (11), *lsrK* (encoding a kinase responsible for AI-2 phosphorylation) (12), and *lsrR* (encoding a repressor for the *luxS*-regulated *lsr* operon) (13). However, there was no obvious difference in the transcription of *lsrB*, *lsrK,* and *lsrR* between the wild type and the *luxS* mutant, which indicates that the genes reported to relate to the recognition and modification of AI-2 molecules may not be applicable to *Av. paragallinarum*. Additionally, as LuxS and Pfs are the only two essential enzymes in the biosynthetic pathway for AI-2 (9), the significantly increased transcription of *pfs* in the *luxS* mutant indicates that LuxS may play an important role in the activated methyl cycle pathway of *Av. paragallinarum*. Together, these findings indicate that LuxS plays an essential role in the production of AI-2 in *Av. paragallinarum* and may also be involved in the activated methyl cycle pathway in *Av. paragallinarum*.

The *luxS* deficiency can reduce the ability of some bacteria to survive in macrophages, indicating that *luxS* plays an important role in immunological evasion after invasion (36, 37). We, therefore, evaluated the ability of wild-type and *luxS* mutant *Av. paragallinarum* to invade HD11 and DF-1 cells. We noted that the deletion of *luxS* resulted in a significant decrease in cell invasion. This showed that the absence of *luxS* may decrease the ability of *Av. paragallinarum* to survive in macrophages and that *luxS* deficiency has an adverse effect on the invasion of the pathogen. Bacterial invasion is an essential factor in bacterial infection and pathogenicity, however, the mechanism by which LuxS affects *Av. paragallinarum* invasion is not clear, and further studies are warranted.

In conclusion, this study showed that LuxS may not only be associated with the activated methyl cycle but is also involved in the AI-2 quorum sensing pathway in *Av. paragallinarum*. Additionally, the *luxS* mutant shows significantly decreased invasive capacity but is not essential for the virulence of *Av. paragallinarum*. Further research on the quorum-sensing system of pathogens may provide novel therapeutic strategies to prevent and control *Av. paragallinarum* in poultry.

## Data availability statement

The datasets presented in this study can be found in online repositories. The names of the repository/repositories and accession number(s) can be found in the article/supplementary material.

## Ethics statement

The animal study was approved by the Beijing Academy of Agriculture and Forestry Sciences Animal Care and Use Committee. The study was conducted in accordance with the local legislation and institutional requirements.

## Author contributions

DL: Data curation, Formal analysis, Methodology, Writing – original draft. CH: Data curation, Formal analysis, Funding acquisition, Writing – review & editing. GL: Investigation, Resources, Writing – original draft. MZ: Investigation, Resources, Writing – original draft. FX: Resources, Writing – original draft. JQ: Conceptualization, Funding acquisition, Supervision, Writing – original draft, Writing – review & editing. HS: Conceptualization, Funding acquisition, Methodology, Supervision, Writing – original draft, Writing – review & editing.
