# Supplementary figures and images for: The absence of luxS reduces the invasion of Avibacterium paragallinarum but is not essential for virulence
Source: Front Vet Sci. 2024 Aug 27;11:1427966. doi: 10.3389/fvets.2024.1427966 (PMC11390136; doi:10.3389/fvets.2024.1427966)

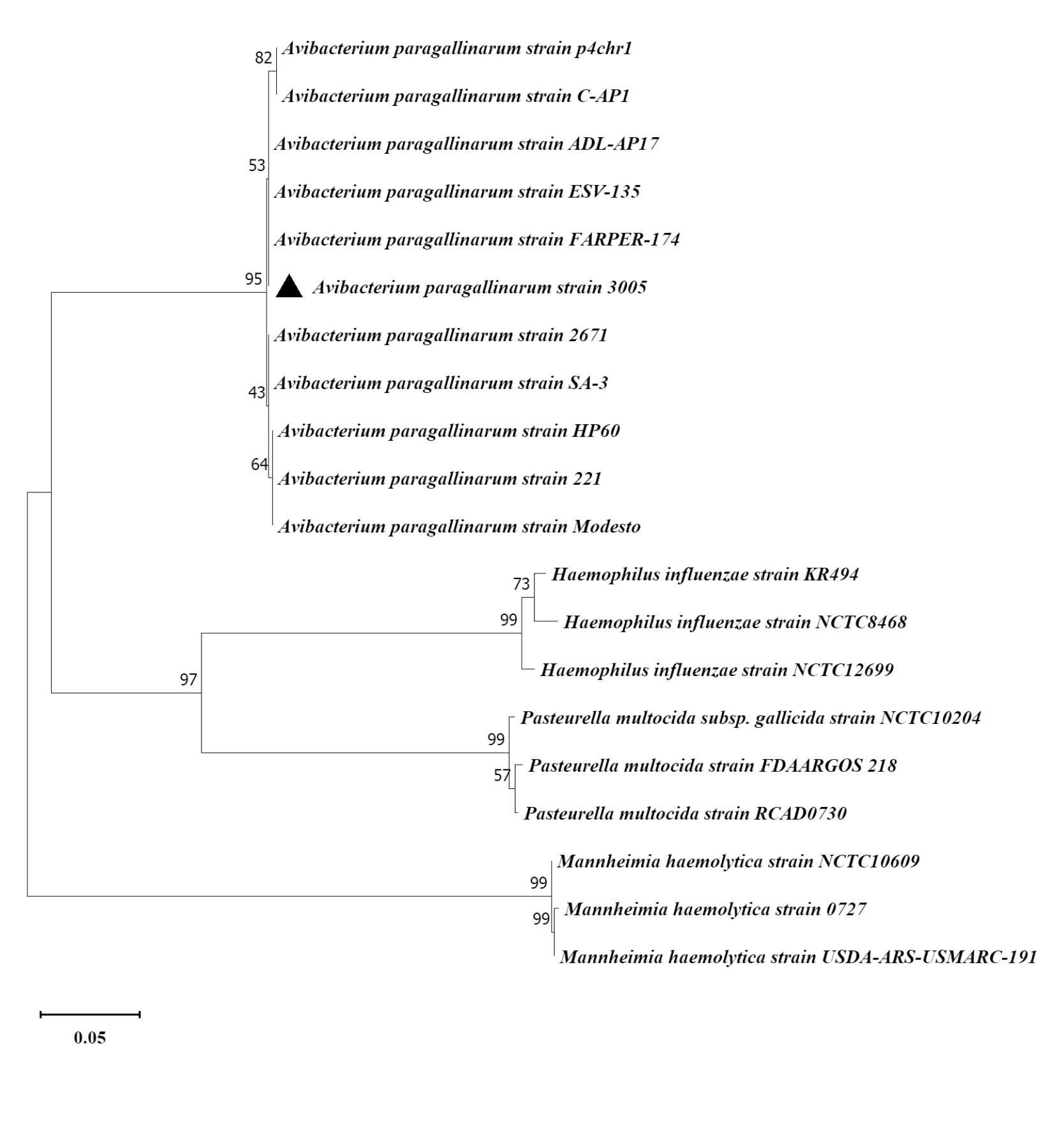

Supplement: Supplementary file 1 [file Image_1.tif]
